# Combining Community Engagement and Scientific Approaches in Next-Generation Monitor Siting: The Case of the Imperial County Community Air Network

**DOI:** 10.3390/ijerph15030523

**Published:** 2018-03-15

**Authors:** Michelle Wong, Esther Bejarano, Graeme Carvlin, Katie Fellows, Galatea King, Humberto Lugo, Michael Jerrett, Dan Meltzer, Amanda Northcross, Luis Olmedo, Edmund Seto, Alexa Wilkie, Paul English

**Affiliations:** 1 California Environmental Health Tracking Program, Public Health Institute, 850 Marina Bay Parkway P-3, Richmond, CA 94804, USA; galaking22@gmail.com (G.K.); DanMeltzer@gmail.com (D.M.); alexa.wilkie@phi.org (A.W.); 2 Comite Civico del Valle, 235 Main St, Brawley, CA 92227, USA; esther@ccvhealth.org (E.B.); humberto@ccvhealth.org (H.L.); luis@ccvhealth.org (L.O.); 3 Department of Environmental and Occupational Health Sciences, University of Washington, Chair’s Office F463, Box 357234, Seattle, WA 98195-7234, USA; gncarvlin@gmail.com (G.C.); fellowsk@uw.edu (K.F.); eseto@uw.edu (E.S.); 4 UCLA Fielding School of Public Health, University of California Los Angeles, 650 Charles E. Young Drive South, 56-070B CHS, Los Angeles, CA 90095, USA; mjerrett@ucla.edu; 5 Department of Environmental and Occupational Health, George Washington University, 950 New Hampshire Ave. NW, 4th Floor, Washington, DC 20052, USA; northcross@email.gwu.edu; 6 California Department of Public Health, 850 Marina Bay Parkway P-3, Richmond, CA 94804, USA; paul.english@cdph.ca.gov

**Keywords:** air monitors, community air monitoring, sensors, community-engaged research, air quality, particulate matter, citizen science

## Abstract

Air pollution continues to be a global public health threat, and the expanding availability of small, low-cost air sensors has led to increased interest in both personal and crowd-sourced air monitoring. However, to date, few low-cost air monitoring networks have been developed with the scientific rigor or continuity needed to conduct public health surveillance and inform policy. In Imperial County, California, near the U.S./Mexico border, we used a collaborative, community-engaged process to develop a community air monitoring network that attains the scientific rigor required for research, while also achieving community priorities. By engaging community residents in the project design, monitor siting processes, data dissemination, and other key activities, the resulting air monitoring network data are relevant, trusted, understandable, and used by community residents. Integration of spatial analysis and air monitoring best practices into the network development process ensures that the data are reliable and appropriate for use in research activities. This combined approach results in a community air monitoring network that is better able to inform community residents, support research activities, guide public policy, and improve public health. Here we detail the monitor siting process and outline the advantages and challenges of this approach.

## 1. Introduction

More than 3 million people worldwide die prematurely every year as a result of outdoor air pollution [1]. In particular, exposure to particulate matter (PM) has been found to be associated with an increased risk of mortality and excess hospitalization even at levels below regulatory limits [2,3]. While governmental regulatory air monitoring plays an essential role in achieving air quality goals, the monitors used are expensive and require a high degree of training to operate and maintain. The resulting data typically have low geospatial resolution due to the sparseness of monitors, thus limiting their utility for understanding real-time, local-level air quality conditions. While the application of spatial interpolation techniques to regulatory monitoring data has been used to estimate air quality in locations without monitors [4,5], a greater number of air monitors distributed throughout an area of concern will improve a model’s utility for identifying air pollution hot spots and characterizing local community exposures. Finally, despite being generated by high-quality monitors using federally-approved methods, regulatory air monitoring data may not be relevant, trusted, or understood by residents.

With the increasing availability and quality of small, low-cost air sensors, many public health and research projects are now employing next-generation air monitoring technology to conduct personal and local-level air monitoring [6,7,8]. While not approved in the United States for use in regulatory monitoring, this new technology holds great potential for addressing gaps in regulatory air monitoring data to better characterize air quality at the community level. Yet, few projects have attempted to establish a permanent community air monitoring network that produces data that will address community information needs and support scientific research.

By involving residents in decision-making, conducting transparent and inclusive activities, demystifying scientific processes, integrating community knowledge, and facilitating relationship-building, a community-engaged approach to research and other data collection activities can increase community trust, understanding, and use of the resulting data [9]. As applied to air quality monitoring, a community-engaged approach may involve communities in determining research topics and study design, collecting data, conducting analysis, and interpreting and disseminating results to improve environmental and health outcomes. Early examples of community engagement in air monitoring involved grab sampling—taking a single sample within a short period of time—and were not intended for sustainable and continuous public health surveillance. There are also many examples of communities participating in the initiation, design, and/or implementation of efforts to monitor air pollution near specific sources, also known as fenceline monitoring [10,11,12].

However, apart from a few case studies, communities have not traditionally been engaged in the design of community air monitoring networks, defined here as the distributed installation of monitors to measure ambient air quality levels within geographic communities. Instead, community air monitoring networks are often developed by siting monitors at locations of convenience or in locations selected without community consultation, resulting in monitoring data that are of limited utility for researchers or residents, respectively [13].

The siting of monitors in a network has important implications for the utility of the resulting data. Broadly speaking, data from monitors placed where people live and are most concerned about air quality are most useful for community needs, while data from monitors that are spread out and placed in a variety of land uses are most useful for modeling the spatial pattern of air quality using land use regression methods [14]. Arguably important for both uses, data quality can be assured by incorporating air monitoring best practices, such as validation, quality assurance, and quality control procedures. However, to the authors’ knowledge, there has not been a community air monitoring network implemented at a large geographic scale that successfully addresses these diverse priorities, until now.

### The Imperial County Community Air Monitoring Project

Imperial County is home to a primarily Latino population (84%) and has some of the highest rates of unemployment (47%) and poverty (24%) in the nation [15]. The county is primarily a desert ecosystem, much of which has been converted to agricultural land. The county has a range of air pollution sources that contribute to regular and sustained exceedances of the California PM standards [16,17], including nearly 8 million vehicles annually crossing the U.S.-Mexico border in Calexico [18], an average of about 28,000 acres of agricultural field burned annually [19], and the drying Salton Sea [20].

Exposure to PM is related to increased respiratory disease, decreased lung function, and increased asthma attacks in susceptible individuals [21]. Short-term exposure to high levels of PM is related to increased heart attacks, while long-term exposure to PM is related to increased heart disease and premature mortality [22,23]. Imperial County has the highest rate of both emergency visits and hospitalizations for asthma among school-age children in California [24]. Additionally, Imperial County ranks among the top three counties in the state for hospitalizations from heart attacks [25].

Imperial County has only five regulatory PM monitors to cover its 175,000 residents and over 4400 square miles (an area nearly the size of Connecticut). This regulatory network does not allow many residents to understand their local air quality, and its data are of limited use in identifying air pollution hotspots or examining other local trends. Furthermore, there has been historical mistrust of government air quality data by the community, primarily driven by observed disconnects between the reported air quality and what residents experience, compounded by a perceived lack of access to the data. Examples shared by community members include the standard practice of removing data for high pollution “exceptional events” (such as fires) from regulatory datasets, as well as reported instances when regulatory monitors were offline or did not report very high PM levels when the air quality at those monitoring locations was poor enough to obscure visibility.

To meet community needs for relevant, trusted, community-level air quality data, the California Environmental Health Tracking Program (CEHTP) and its main project partners—the Imperial County community-based organization Comite Civico del Valle (CCV) and the University of Washington (UW)—initiated the Imperial County Community Air Monitoring Project [26]. Funded by the National Institute of Environmental Health Sciences (NIEHS) Research to Action program, the project utilized community engagement and participation approaches and air monitoring best practices to establish a community air monitoring network (CAMN) of 40 PM monitors in Imperial County, California. The goal of the network was to provide residents with accurate, real-time data on air quality in their communities that could also be used in scientific analysis to identify local trends and hotspots. This article focuses on the project’s innovative monitor siting methodology, which utilized a phased approach that integrated a community process to identify and select monitor sites, a monitor calibration and validation process, and spatial analysis as mechanisms to integrate community and scientific priorities into the design of the network.

## 2. Materials and Methods

### 2.1. Community Engagement Structure

In order to ensure meaningful community participation, the first step was to design a community engagement structure to guide the project. CCV’s co-investigator role and active participation in project initiation provided a strong community foundation. Next, the project’s Community Steering Committee (CSC)—consisting of 19 community members that included local advocates, concerned residents, and youth—contributed additional guidance, participation, and decision-making in key activities throughout the project. Finally, a broader group of residents was convened to contribute their community-specific knowledge and perspectives to the project, including the site selection process. Other stakeholders were engaged through a technical advisory group comprised of air monitoring experts from the local air district, the California Air Resources Board (CARB), the US EPA, and other agencies. More in-depth technical guidance was provided by academic consultants from the University of California at Los Angeles and George Washington University.

### 2.2. Deployment of Monitors in Stages

**Figure 1:**
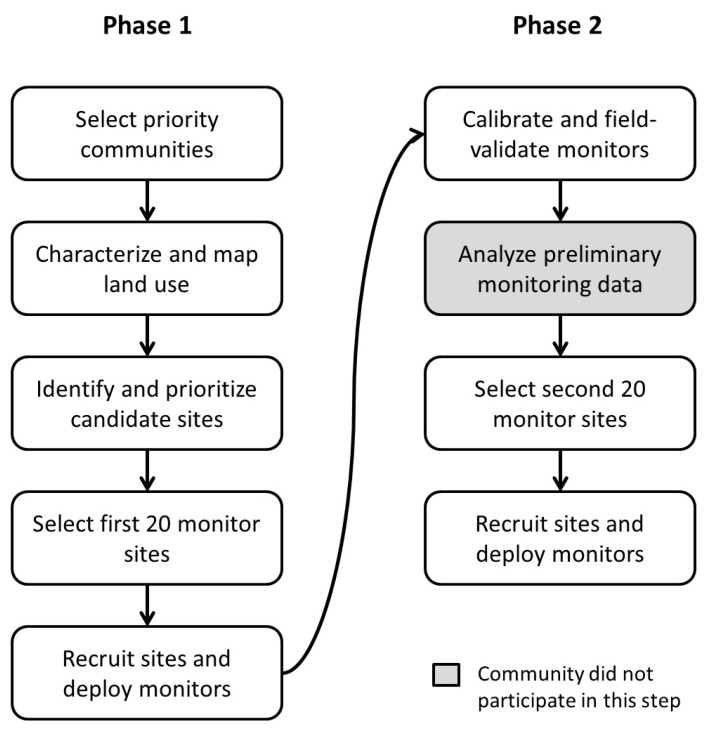
Diagram of the participatory process to site monitors.

The project team used a staged approach to deploy the monitors (see Figure 1). In Phase 1, the first set of 20 monitors was deployed at sites selected through a community process. In Phase 2, a second set of 20 monitors was then deployed based on the results of preliminary monitoring data from Phase 1. This staged approach ensured that community priorities for monitoring locations could be met in Phase 1, and that Phase 2 could be used to fill in any geographic gaps in data collection to ensure scientific integrity of subsequent spatial modeling conducted with the data.

### 2.3. Phase 1: Community Process to Select the First Set of Monitoring Sites

Selection of priority communities. In May 2014, to begin the monitor siting process, the CSC identified communities (i.e., towns and cities) in which monitor placement would be a priority due to the vulnerability of the residents to air pollution. First, the CSC reviewed county-specific maps and tables of various environmental, health, and social indicators, and considered factors that impact personal and community vulnerability to air pollution. The CSC then used ranked voting to select the final 11 communities.

Land use characterization. To ensure that data from the Phase 1 monitors sites could be used to model PM concentrations (to inform Phase 2), it was important that the first set of sites represent diverse land uses and potential air pollution sources. Environmental priorities related to air pollution were identified and ranked by the CSC. Based on this prioritization, researchers compiled spatial data on agricultural land uses, agricultural burning, stationary air emissions, slaughterhouses, feedlots, crematoriums, roadway proximity, Salton Sea proximity, railroads, solar plants, vehicle emissions, vehicle-related dust (e.g., off-roading), and wind turbines. The CSC also provided input on community vulnerability factors, and spatial data on demographic variables and health outcomes, such as asthma, were compiled as well.

A principal component analysis (PCA) was then conducted to statistically determine combinations of variables that accounted for the majority of variance in the compiled land use data. From the analysis, 10 principal components were identified, and corresponding regions within Imperial County were mapped. For Phase 1, it was required that at least one monitor be placed in each PCA region so that the network would collect air quality data from the broadest number of land uses.

Identification and prioritization of candidate sites. In January 2015, 45 residents from the 11 priority communities were recruited to participate in a two-day process to identify and prioritize candidate air monitoring sites in their communities. On the first day, participants learned about air monitoring, uses of air quality data, and considerations for monitor siting. Participants were grouped by community to (1) review maps of their community; (2) consider who in their community is most vulnerable to air pollution, where an air monitor would be most useful, and how the monitoring data might be used; and (3) create a list of candidate air monitoring sites based on these considerations. Participants were not limited in the number of sites they could identify, and consensus was not required for a site to be considered a candidate. Participants were asked to ensure that at least one candidate site was located in their community’s priority PCA region.

**Figure 2:**
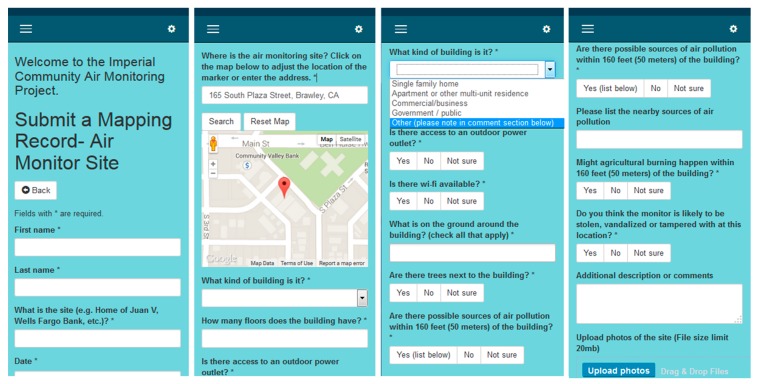
Screenshots of mobile web form used for data collection.

The following day, the community participant groups visited their respective candidate sites. With training from the previous day, groups assessed site characteristics (such as building height, security, likelihood of available Wi-Fi and AC power supply, and locations of nearby air pollution sources), took photos or videos of the site using a mobile device, and reported this information on a custom-designed mobile web form (see Figure 2) modified from IVAN Imperial, CCV’s existing community environmental reporting website [27]. Smartphones were provided when needed, along with paper forms as back-ups. Each group submitted one mobile web form per site. Once all forms were completed, the participant groups reviewed their reports and selected three priority monitor sites for their community.

Selection of Phase 1 monitoring sites. Of the 20 monitors to be deployed in Phase 1, one was set aside for colocation with a state regulatory monitor (operated by CARB) and another was colocated with a non-regulatory Imperial Irrigation District (IID) monitor to allow researchers to assess data quality of the CAMN instruments. Sites for the remaining 18 monitors were selected by the project team from the entire list of candidate sites and were guided by two requirements. First, at least one monitor should be placed in each of the 11 priority communities, with the larger communities of Brawley, Calexico, El Centro, and Imperial receiving at least two monitors. This was to ensure that each priority community would have community-specific data. Second, each of the 10 PCA region types should be represented at least once in the final set of 20 monitors. This was to ensure that monitoring data could be used to conduct a land use regression in Phase 2. Using these criteria and a process of elimination that favored installing monitors in at least one priority site per community, 18 sites were identified, each with two alternatives. UW and UCLA provided suggestions about the spatial distribution of the monitors, while CCV provided insights into the feasibility of obtaining agreements to install monitors at the various sites.

Site recruitment. Recruitment of air monitoring sites began in March 2015. Site-specific contact methods were used, such as formal letters, introduction through a trusted intermediary, or a cold visit. Multiple meetings, often with different individuals (e.g., school principal and maintenance staff), were required for each site. A factsheet was provided to each site describing the project, the air monitors, and the requirements and benefits of hosting a monitor. CCV then co-signed a form with each site representative that confirmed permission for CCV staff to install a monitor at the location and, with reasonable notice, gain access to the monitor to perform maintenance and repairs. If a site declined participation, an alternate site was contacted.

Deployment of Phase 1 monitors. A Dylos 1700 (Dylos Corporation, Riverside, CA, USA) laser particle counter was used for the project, and modified to include four size bins (>0.5 µm, >1 µm, >2.5 µm, >10 µm). In addition to the Dylos sensor, the monitors were customized with additional equipment to enable wireless Internet connectivity and measurements of temperature and humidity. The monitors included a protective shelter that does not inhibit measurements, along with a cooling fan that turns on when the external temperature reaches 120 °F [28]. At the sites, monitors were installed on rooftops or the unobstructed sides of buildings, higher than 1 meter and lower than 14 m above the ground. Monitors were connected to AC power and to the Internet via that site’s own internet service or, in Phase 2, with mobile internet hotspots. Data were delivered via the Internet to UW servers.

### 2.4. Phase 2: Selection of Monitoring Sites Based on Data Analysis

Monitor calibration and field validation. To ensure data quality, each of the monitors was calibrated and field validated against both PM_2.5_ and PM_10_ federal equivalent method (FEM) beta-attenuation monitors (BAMs) and federal reference method (FRM) gravimetric filters at a colocation site in the study area. A conversion equation was developed to estimate particle mass concentrations from the native Dylos particle counts taking into account relative humidity [28].

**Figure 3:**
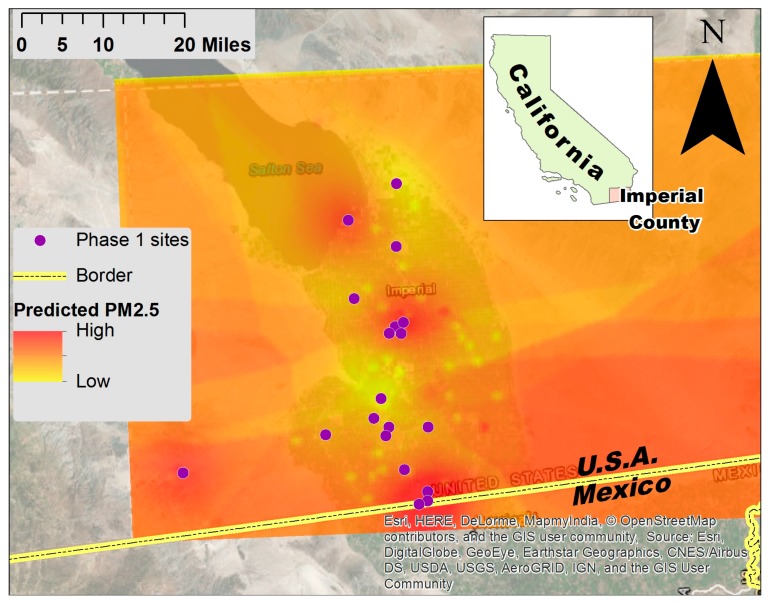
Modeled air pollution concentrations. Purple dots indicate locations of Phase 1 monitors.

Spatial analysis of preliminary air quality data. To determine where Phase 2 monitors should be placed, a preliminary map of air pollution concentrations across the valley was developed by a spatial interpolation Kriging model using the concentrations measured at 19 sites over a 52-day period ending in February 2016 (Figure 3).

A land use regression model was created to predict PM_2.5_ based on land use, meteorological, and temporal variables. PCA was used to reduce the number of land use variables. The first 10 principal components explained 95% of the variance. We used those 10 principal components along with temperature, relative humidity, wind direction, wind speed, distance to the US-Mexico border, and distance to the Salton Sea. These explanatory variables were put into a linear model with hourly PM_2.5_ as the response variable. This model was then used to predict PM_2.5_ on a grid of points placed every 250 m across Imperial County. All of the hourly predictions were averaged across the length of the study. The resulting spatial differences in predicted PM_2.5_ helped inform the site selection process. After identifying locations without monitors where additional monitoring data would help to better characterize air quality (e.g., locations where modeled concentrations appeared to change drastically over a small geographic area), 20 general locations for the Phase 2 monitors were proposed.

Site selection and deployment of Phase 2 monitors. In May 2016, the project team and CSC members examined the proposed Phase 2 monitor locations using satellite imagery on Google Maps to identify potential monitor sites. Due to the limited number of buildings at these rural locations, a formal site selection process was not used. Instead, CCV and interested CSC members visited these locations to identify and recruit monitoring sites, ideally within a two-mile buffer of the proposed location. If sites within the buffered area could not be identified, or declined participation, the monitor was deployed at a site in an alternate location identified by the project team. The deployment of the second set of monitors was completed by April 2017, with data automatically transmitted from the monitors to UW servers via the Internet.

## 3. Results

### 3.1. Sites Selected through the Phase 1 Community Process

The aim of the Phase 1 process was to place monitors in locations of relevance to the community, such as places where groups vulnerable to air pollution (e.g., children, elderly) spend time, places where residents are likely to experience poor air quality, or places that are well-known or otherwise meaningful to community members. During the community monitor site selection process, participants identified a total of 85 candidate sites in 11 communities. From these, they selected 33 priority sites (three per community), classified as schools (12), government buildings (7), businesses (7), non-profit organizations (4), residences (2), and a park. Twelve of these were chosen during the site selection process, as described previously, to be included in the group of 20 Phase 1 sites.

Upon recruitment, two schools declined participation due to concern that having an air monitor would cause worry among parents or create stigma that could impact enrollment. Two other selected sites could not be used because they lacked Internet services, which was still a siting requirement at the time of recruitment. In place of these, four alternate sites were recruited to host a monitor. Due to the widespread preference among the community participants and within CCV toward school sites, the project team chose schools as alternates.

Ultimately, the Phase 1 monitors were deployed at 14 public schools (including one colocated with a state regulatory monitor), two government buildings, a non-profit organization, a residence, a business, and a national wildlife refuge near the Salton Sea (colocated with an IID monitor). Of the 20 monitors, 19 were placed within the priority communities identified by the CSC. Of these, 10 monitors were installed at sites prioritized by community participants, and two monitors were installed at alternate sites that were chosen due to the proximity to their original priority sites.

The monitors were placed in nine out of the 10 PCA regions identified early in Phase 1. The remaining PCA region corresponded entirely to a state prison. Due to the lack of response to initial inquiries and concerns among the project team about the feasibility of getting permission in a timely manner, an exception was made to the requirement to site a monitor in each PCA region. The prison was not included as one of the original 20 selected sites, and this decision was supported by the CSC.

Feedback from community participants during community meetings and through meeting feedback forms indicated satisfaction with the process and final sites selected. The only disappointment expressed was related to the two schools that declined participation.

### 3.2. Calibration and Validation

While the main purpose of Phase 1 was to focus on community-relevant monitor siting, it was also a stated priority among both the project team and CSC to ensure that the CAMN data were considered valid and useful by scientific researchers. By colocating the CAMN monitors with the regulatory monitor, project researchers were able to use data from both to assess, calibrate, and field-validate the CAMN monitors. This was an important step to ensuring data quality, and results indicated that the CAMN monitors generally performed well.

Briefly, we found that the R^2^ between converted hourly averaged Dylos mass measurements and a PM_2.5_ FEM BAM was 0.79 and PM_10_ FEM BAM was 0.78. The performance of the conversion equation was evaluated at six other sites with colocated PM_2.5_ environmental beta-attenuation monitors (EBAMs) located throughout Imperial County. The agreement of the Dylos with the EBAMs was low to high (R^2^ = 0.35 to 0.81). More details can be found in the published results [28].

The colocations also allowed project researchers to develop an algorithm to convert the CAMN data from particle count to particle mass concentrations [28], a more commonly-used measurement of PM that would be more relevant for communicating results to the community.

### 3.3. Sites Selected Based on Phase 2 Spatial Analysis

**Figure 4:**
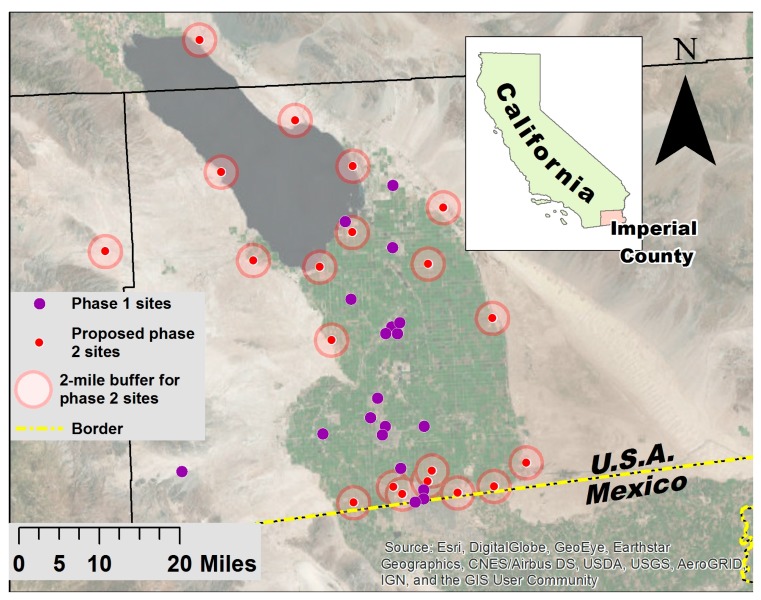
Proposed locations for Phase 2 monitors identified based on preliminary spatial analysis.

The aim of the Phase 2 process was to deploy monitors in areas to improve the network’s ability to provide data useful for identifying trends and hotspots. Spatial interpolation of preliminary data from the Phase 1 monitors was used to develop a preliminary map of modeled PM_2.5_ concentrations in order to assess locations where more monitoring data would be useful. The results indicated a need to better characterize gradients of pollution coming from the eastern and western mountains of the valley, from the Salton Sea, and from the U.S.-Mexico border (Figure 4).

Using the Phase 2 site selection process described previously, six monitors were deployed at the Salton Sea, with three additional monitors placed farther to the south and east. Another seven monitors were placed along the U.S.-Mexico border, including one site in Mexicali, Mexico. Two additional monitors were placed to the east and in the center of the populated region of the county, at the agricultural-desert and agricultural-urban interface, respectively. The final two monitors were installed at the original colocation sites from Phase 1 to facilitate further assessment of monitor performance and data quality.

The new sites were primarily private residences (12), with colocations with IID monitors (5), and a government building. Based on lessons learned from the operation of the Phase 1 monitors, all monitors in Phase 2 were outfitted with a cellular hotspot for internet connectivity if a site lacked reliable Internet services.

### 3.4. Completed Network Provides New, Locally-Relevant Information

**Figure 5:**
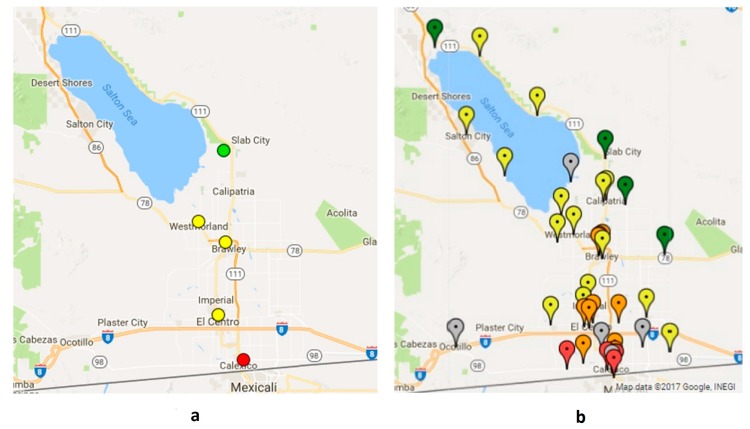
Screenshots of online maps showing air monitor locations and real-time air quality readings taken from (**a**) the regulatory network website and (**b**) the Imperial County CAMN website, on 20 December 2017 [29,30]. For both maps, the color of the monitor markers correspond to health risk related to current air quality conditions, where green is lowest risk, yellow is moderate risk, orange is unhealthy for sensitive populations, and red is unhealthy. Gray markers on the CAMN map indicate monitors that are offline.

The goal of the project was to establish a CAMN that would provide residents with accurate, real-time data on air quality in their communities that could also be used in scientific analyses to identify local trends and hotspots. The process to deploy all 40 monitors of the CAMN took about three years in total, with significant community engagement throughout. Upon completion of Phase 2, the CAMN consisted of 40 monitors, many in communities that previously lacked local air quality data due to their distance from a regulatory air monitor (Figure 5).

Specifically, CAMN monitors are now located within 13 cities and towns in Imperial County (excluding monitors sited in communities located adjacent to the county boundaries in Mexico and Riverside County), compared to the five in which regulatory monitors are located. With eight times the number of monitors as the regulatory network, the CAMN also has a greater monitor density than the regulatory network. These factors result in greater coverage of populated areas by the CAMN, compared to the regulatory monitors.

The real-time CAMN data have been made publicly available through CCV’s IVAN Imperial Air Monitoring website [29]. An interactive web map showing real-time data for each site, along with summary data for individual monitors, is available on the website. As demonstrated in Figure 5, the mapped real-time CAMN data provide more insight into the spatial distribution of PM compared to the real-time data from regulatory monitoring alone.

Preliminary results of land use regression modeling (distinct from the analysis described above) conducted by the authors with the CAMN data also showed gradients of exposure, temporal patterns, and hotspots. Comparisons with regulatory monitors also demonstrate that the CAMN captured more episodes of elevated PM levels than regulatory monitors. The results from both analyses are currently being submitted for publication.

### 3.5. Initial Response to the Community Air Monitoring Network

In discussions and meeting evaluations, community participants and partners overwhelmingly expressed satisfaction in the site selection process and the final monitor sites. They also stated an intention to use data from the CAMN and believed the data would be trustworthy. They attributed this to their participation in the project activities, increased understanding about air monitoring, and engagement in project decision-making processes.

While outreach activities are ongoing to inform county residents about the CAMN, these data are already being used on a daily basis by eight of the school sites to alert them when PM levels are high enough to keep asthmatic students indoors or, if higher, implement a rainy day schedule schoolwide. School staff access the CAMN data via the public website and via email alerts. While some of these schools had policies in place regarding air quality and began using the CAMN as an additional source of data, others initiated these policies as a direct result of having a monitor located on their campus.

The CAMN data have also generated interest beyond the community. For example, CAMN data have been requested by the local public health department to inform the development of the county general plan, by CARB to investigate a PM episode, by a journalist investigating air pollution issues along the US-Mexico border, and by university researchers to enhance the methodology for using satellite data to study ground-level PM pollution.

## 4. Discussion

The novel methodology used to site monitors for this study addresses several barriers that regulatory networks face in meeting community needs. Specifically, regulatory monitoring networks are sparse, often do not have monitors in locations of most relevance to communities, and may not be trusted by community members. The increased availability of small, low-cost air sensors now makes it feasible for researchers to establish denser networks. However, without community input and engagement, the resulting data may still be of limited utility and may lack credibility within the community. Community-led monitoring efforts are more likely to be trusted and relevant for communities; yet these efforts are often under-resourced, with limited access to scientific expertise or conventional monitoring equipment for colocations, resulting in monitoring data that may not achieve the level of scientific rigor or research utility that the community desires. 

Our siting methodology is distinguished by the deliberate integration of community and scientific priorities. In fact, the conceptual separation of these priorities did not match our experience. Community participants consistently emphasized the importance of scientific quality, while a primary goal among research partners was to ensure that the CAMN met community needs.

Results suggest that a CAMN developed using this study methodology can provide scientifically rigorous, community-relevant air quality data to complement the regulatory networks. The provision of real-time data at community-relevant locations means that individuals can use CAMN data to change behaviors to reduce PM exposures, while the ability to identify temporal and spatial pollution patterns increases the utility of the data for public health planning and policymaking at community and regional levels. As the next generation of small, low-cost air sensors offers new opportunities for establishing CAMNs, it is important to continue developing and evaluating siting approaches with respect to their ability to promote data quality and relevance for communities and researchers alike.

There may be limitations in the utility of the study methodology for other communities, in which residents may be concerned about different air pollutants, specific sources, or other community-specific factors. Furthermore, limited access to the time, resources, or required skillsets may hinder close replication of this process. However, it may be possible to customize or scale this process while retaining key aspects of the methodology. For example, meaningful community engagement can happen in many ways; community residents need not visit nor collect data on candidate sites in order to identify and prioritize them. Comprehensive monitor calibration and validation activities may not be needed if the selected instrument has already been rigorously tested in lab and field settings. The two-part siting approach, which prioritizes monitor placement at community-relevant sites, also provides flexibility by allowing a phased deployment of the CAMN.

While not directly related to monitor siting decisions, any efforts involving the long-term deployment of community monitors must take into consideration potential challenges, costs, and staffing requirements to maintain, repair, and replace monitoring equipment. Similarly, it is also important to acknowledge that effective outreach and communication plays a critical role in the overall utility of the CAMN, and data must be displayed, interpreted, and made accessible in a community-appropriate and scientifically-accurate manner. As part of the broader project, the authors have undertaken efforts to address these two issues [26].

Finally, this work can inform efforts to better integrate CAMNs with government air monitoring activities, particularly as agencies explore the use of small, low-cost air sensors to address data needs that cannot be met by the existing regulatory networks [31,32,33]. In California, recent legislation (California Assembly Bill 617) requires state and local regulatory agencies to install community air monitoring systems in priority communities [34]. In response, the Imperial County Community Air Monitoring Project and its CAMN (called “IVAN AIR”) have been examined by state legislators as “a potential model for future monitoring and air quality work across the state” [35] and by CARB as one of the models that will inform their AB 617 implementation plan [36].

## 5. Conclusions

Community air monitoring networks that utilize small, low-cost air sensors can produce data that complement regulatory networks, which are often insufficient for community needs. However, careful consideration should be made with regard to monitor locations, which fundamentally impact the utility of the resulting data.

To address the need for more air monitoring data within communities of concern throughout Imperial County, we used a unique monitor siting methodology that addressed scientific and community priorities. Key aspects of the approach included the calibration and field-validation of the monitors and the deployment of monitors in community-identified sites, among different land uses, and in locations to better characterize gradients of PM concentration.

Using this community-engaged approach, we deployed a CAMN of 40 PM monitors throughout Imperial County. The resulting data are being used to inform exposure reduction behaviors and to conduct research analyses, and they have a high potential to inform public health actions and policies. 

Strong community partnerships and meaningful engagement was critical to the success of this process, which has generated attention from communities and government agencies nationally. As next-generation air monitoring technology garners interest from communities, researchers, and government, this methodology may serve as a model for the collaborative, community-driven development of community air monitoring networks.
